# Success or Failure? Are We Meeting the Needs of Children With Developmental Coordination Disorder?

**DOI:** 10.1177/00084174231197618

**Published:** 2023-09-06

**Authors:** Erin S. Klein, Melissa Licari, Skye Barbic, Jill G. Zwicker

**Keywords:** Developmental coordination disorder, Motor skills disorder, Rehabilitation, School, Therapy, Trouble développemental de la coordination, trouble de la motricité, école, réadaptation, thérapie

## Abstract

**Background.** Current international clinical practice guidelines indicate that children with developmental coordination disorder (DCD) should receive therapy, yet school and community-based occupational therapy is not standard of care. **Purpose.** To understand parent perspectives on best practice for treatment and what supports and services are required to meet their children's needs. **Method.** An online cross-sectional survey (impACT for DCD) was distributed to parents of children <18 years with self-reported suspected or diagnosed DCD living in British Columbia. Data analysis included descriptive statistics and contingency analyses to explore whether access to therapy differed with income, age of child, or geographical location. Open-ended questions were analyzed using content analysis. **Findings.** Of the 237 respondents, 194 children had suspected/confirmed DCD; however, only 20% (38/198) of the children had received therapy at school. Some parents (32/58) pursued private therapy. Geographic location and income had no relationship with therapy access (*p* > 0.05). Parents expressed frustration with poor awareness and understanding of the impact of DCD among educators, health-care professionals, and community members, and identified the need for funded and accessible school and community services and supports. **Conclusion.** Evidenced-based occupational therapy intervention should be standard of care for children with DCD as per clinical guidelines and parent-identified need.

## Résumé

**Description.** Les lignes directrices internationales actuelles en matière de pratique clinique indiquent que les enfants souffrant d'un trouble développemental de la coordination du développement (TDC) devraient bénéficier d'une thérapie, mais l'ergothérapie à l'école et dans la communauté n'est pas la norme en matière de soins. **But.** Comprendre le point de vue des parents sur les meilleures pratiques de traitement et sur les soutiens et services nécessaires pour répondre aux besoins de leurs enfants. **Méthodologie.** Une enquête transversale en ligne (impACT for DCD) a été distribuée aux parents d'enfants de moins de 18 ans vivant en Colombie-Britannique et dont le TDC est soupçonné ou diagnostiqué. L'analyse des données comprenait des statistiques descriptives et des analyses de contingence pour déterminer si l'accès à la thérapie différait en fonction du revenu, de l'âge de l'enfant ou de la situation géographique. Les questions ouvertes ont été analysées à l'aide d'une analyse de contenu. **Résultats.** Sur les 237 personnes interrogées, 194 enfants présentaient un TDC suspecté/confirmé ; cependant, seuls 20 % (38/198) des enfants avaient bénéficié d'une thérapie à l'école. Certains parents (32/58) ont suivi une thérapie privée. La situation géographique et les revenus n'avaient aucune relation avec l'accès à la thérapie (p>0,05). Les parents ont exprimé leur frustration face au manque de sensibilisation et de compréhension de l'impact du TDC parmi les éducateurs, les professionnels de la santé et les membres de la communauté, et ont identifié le besoin de services et de soutiens financés et accessibles à l'école et dans la communauté. **Conséquences.** L'intervention ergothérapeutique fondée sur des données probantes devrait être la norme de soins pour les enfants atteints de TDC, conformément aux lignes directrices cliniques et aux besoins identifiés par les parents.

## Introduction

Developmental coordination disorder (DCD) is a neurodevelopmental disorder characterized by significant difficulty in learning motor skills and performing daily tasks, such as using cutlery, tying shoelaces, printing, or riding a bicycle. DCD affects 5% to 6% of school-age children, which equates to one to two children in every Canadian classroom (American Psychiatric Association, 2013; Missiuna et al., 2012). Children with DCD can have gross and/or fine motor challenges, with motor performance that is typically slower, less accurate, and more variable compared to their peers (Zwicker et al., 2012). These challenges tend not to improve with age; rather, there appears to be a cumulative effect of the negative developmental experiences impacting both child and family (Jasmin et al., 2018; Missiuna et al., 2007; Stephenson & Chesson, 2008; Summers et al., 2008). Motor coordination challenges can lead to detrimental impacts, with cumulative and chronic manifestations to a child's physical, social, emotional, cognitive, and psychological well-being (Blank et al., 2019; Farmer et al., 2017; Missiuna et al., 2012; Polatajko & Cantin, 2005; Tal-Saban & Kirby, 2018; Zwicker et al., 2013) as well as lower quality of life (Karras et al., 2019; Zwicker et al., 2018).

To mitigate motor coordination challenges and secondary consequences associated with DCD, international clinical practice recommendations state that children need treatment and support at school (Blank et al., 2019). Parents, however, have identified barriers for securing a diagnosis (Missiuna et al., 2006; Novak et al., 2012; Pentland et al., 2016; Soriano et al., 2015; Stephenson & Chesson, 2008; Klein et al., 2023), which is often necessary to access rehabilitative supports and services. DCD is still frequently under-recognized and under-diagnosed (Blank et al., 2012; Camden et al., 2015; Gaines et al., 2008; Maciver et al., 2011; Wilson et al., 2013). Parents struggle to access services and often, service delivery models do not appear to be responsive to children's needs (Camden et al., 2015; Cleaton et al., 2020; Maciver et al., 2011; Rodger & Mandich, 2005). There is a lack of awareness and understanding at schools, with limited access to school-based services and supports (Camden et al., 2019; Jasmin et al., 2018; Maciver et al., 2011; Missiuna et al., 2006; Novak et al., 2012; Rodger & Mandich, 2005; Stephenson & Chesson, 2008). These challenges with accessing rehabilitative and school-based supports speak to the need for a standard of care to ensure efficient, equitable, and accessible service delivery.

Parental experience when navigating healthcare and school systems to access services and supports has been evaluated in other countries and in the province of Ontario using focus groups (Edinburgh, Scotland), interviews (Bristol, England; Ontario, Canada), and survey methodology (Australia) (Licari et al., 2021; Maciver et al., 2011; Missiuna et al., 2006; Novak et al., 2012; Rodger & Mandich, 2005); parents in these studies identified challenges of availability, specificity, and funding to address their child's difficulties associated with DCD. However, there has yet to be a Canadian-based large-scale parent survey that examines the global impact of DCD on the family, specifically the physical, social, emotional, and financial impact of DCD. As healthcare and school funding are within provincial and territorial jurisdiction in Canada (Government of Canada, n.d.), it is important to encapsulate the parental experience unique to the province in which they live. Thus, the objective of this study was to examine the potential barriers and facilitators parents face when attempting to access healthcare and school-based services and supports for their children with DCD in the province of British Columbia. This study will provide a parent perspective on the needed community and school-based services and supports to promote their child's development and participation, information critical to affect provincial policy changes. While the results are specific to British Columbia, the issues are not unique to this province (Camden et al., 2015; Missiuna et al., 2006) or other countries (Licari et al., 2021; Maciver et al., 2011; Novak et al., 2012).

## Methods

### Study Design

This study utilized a cross-sectional survey design to evaluate how parents access rehabilitative and school-based services and support in British Columbia. The project was approved by the University of British Columbia/Children's and Women's Research Ethics Board (H19-00909).

### Participants

The target sample of this study was parents of children under the age of 18 years living in British Columbia with a formal diagnosis of DCD or suspected DCD (with or without common co-occurring conditions, such as attention deficit hyperactivity disorder). Autistic children with functional motor deficits were included given the high co-occurrence of DCD in this population (Kangarani-Farahani et al., 2023; Miller et al., 2021). Parents included biological parents, adoptive parents, and legal guardians. Participants were excluded if: (1) their child's motor coordination challenges were due to a genetic or neurological condition (e.g., Down syndrome, brain injury, developmental delay) as these conditions preclude a diagnosis of DCD; (2) they lacked access to email; or (3) they did not read and understand English.

A multi-pronged sampling strategy was used, which included purposive, convenience, and snowball sampling. Families on the DCD clinic research database and those on the waitlist for a DCD assessment at Sunny Hill Health Centre for Children were sent information about the study and access to an online questionnaire link. We provided information to the College of Occupational Therapists of British Columbia (COTBC), the Physiotherapy Association of British Columbia, the College of Physical Therapists of British Columbia, the College of Speech and Hearing Professionals of British Columbia, the BC Pediatric Society (pediatricians), and child development centers across British Columbia, to target health-care providers that were likely to have contact with parents and children with DCD and were asked to pass along study information to eligible families. Advertisements were posted in community centers, on websites (Zwicker Lab and BCCHR study recruitment), and at BC Children's Hospital and Sunny Hill Health Centre for Children, as well as circulated through social media (Facebook, Twitter, and Instagram). Snowball sampling was also encouraged, so families or professionals could forward the survey link.

### Data Collection

Qualtrics, an online survey management software, was used to create and distribute the survey. The survey was available from October 2019 to April 2020. To prevent respondent duplication, ballot stuffing was enabled, as collection of IP addresses was not permitted by the ethics board. There was an option for parents to provide personal contact information for future research studies; however, this information was not linked to their responses. Post-notification included three reminder emails for those on the recruitment list (sent 2 weeks and then 2 months after the initial email), an additional email reminder to the pediatric OT list-serv through COTBC (sent 1 month after the initial email) and re-posting the survey link on various social media sites (2 weeks and then 1 month after initial posting). Due to COVID-19 provincial lock-downs, additional reminder emails were not sent to the other allied health networks or child development centers.

The impACT for DCD questionnaire was developed in Australia (Licari et al., 2020) to assist with determining the physical, social, emotional, and financial impact of DCD on the child and family. The questionnaire contained 127 open- and close-ended questions, which included questions from the Strengths and Difficulties Questionnaire (SDQ; Goodman, 1997), evaluating the emotional and social impact of DCD. The intent of the questionnaire was also to understand parent-identified supports and services that are beneficial or would be of benefit to mitigate these impacts. Content validity was completed through use of professional and parent focus groups, to ensure that the questions were representative of the issues and concerns from identified stakeholders in the project (parents, medical and rehabilitation professionals, and other researchers. Some modifications were made to the questionnaire used in this study to reflect a Canadian context (winter activity responses, such as skiing) and study aims, but these changes were not substantive (refer to Appendix A for questionnaire). An initial set of 20 completed questionnaires was pre-tested, to evaluate the process of uploading the data and extracting it for analysis. Modifications were made to the questionnaire for improved readability. The survey link was shortened using bit.ly platform, rather than the provided link from Qualtrics, to improve access and usability. Participants indicated their consent through a forced response, located after the consent form, on the first page of the questionnaire. Parents also needed to answer a mandatory question as to whether their child(ren) had motor coordination difficulties to ensure they met the inclusion criteria for participation. Responses to diagnostic questions were also reviewed to ensure children were not diagnosed with another movement-related condition, indicated in the exclusion criteria. To limit non-response bias and increase completion rate, participants had the option to omit questions that may cause undue emotional duress, in addition to skip patterns throughout the survey that direct respondents to only answer those questions that are applicable to them (Draugalis et al., 2008; Evans & Mathur, 2005).

### Data Analysis

IBM^©^ SPSS^©^ version 26 was utilized for descriptive data analysis for quantitative variables from the close-ended questions. Categorical data were analyzed using frequency counts and percentages, while continuous variables were analyzed using medians, interquartile ranges (IQR), and ranges. The Fisher's exact test was used to explore associations between geographic location and access to therapy services, as there was an unequal distribution of data, with some cells of *n* = 0. Independent Mann–Whitney tests were used to explore associations between income and access to therapy. Independent *t*-tests were used to evaluate associations between age and access to therapy. Level of significance was set at *p* < 0.05. To account for multiple analyses, Bonferroni correction was applied.

A complete case analysis/deletion strategy was utilized (Sainani, 2015), with the total number of responses reflected in numerators and denominators to account for missing data. Content analysis (Vaismoradi et al., 2013) was used to describe and contextualize data from open-ended questions using NVivo^©^ software, to expound and understand the quantitative data. Refer to Appendix A for details on survey questions.

## Results

### Response Rates

Three hundred and thirty-six questionnaires were uploaded from Qualtrics. A total of 237 completed and partially completed questionnaires were included in the data analysis after removal of blank questionnaires (*n* = 84) and those that did not meet the inclusion criteria (*n* = 15). Due to snowball sampling and an inability to determine a final count of total survey invitations, it was not possible to calculate a response rate. The completion rate of the study, which measures attrition (Eysenbach & Wyatt, 2002) was 67% (224/336). SurveyMonkey Audience (SMA) has a reported 87% (standard deviation of 10%) to lower than 60% average completion rates (Liu & Wronski, 2018), which is comparable to the completion rate for our study.

### Demographic Data Distribution

Table 1 outlines the demographic distribution of the participants in this study. Most of the children in this sample had a diagnosis for their movement difficulties with 82% (194/237) diagnosed with DCD or another related term (e.g., dyspraxia). With respect to those children with a confirmed or suspected DCD diagnosis and co-occurring conditions, 16% (33/237) had a single diagnosis of DCD, 25% (51/204) had one co-occurring condition, 29% (60/204) had two co-occurring conditions, and 46% (93/204) had three or more co-occurring conditions.

**Table 1:** Participant Characteristics (*N* = 237)

CHILD CHARACTERISTICS	*N* (%)
Age	Median (IQR): 10 (4) years
Male sex assigned at birth	185 (78)
Diagnosis of DCD or similar term (e.g., dyspraxia)	194 (82)
Suspected DCD	43 (18)
Co-occurring conditions (*n* = 204)	
Attention deficit hyperactivity disorder	116 (57)
Learning disorder/disability	132 (65)
Speech/language difficulties	45 (22)
Autism spectrum disorder	51 (25)
Anxiety	106 (52)
Depression	21(10)
Other	48 (23)
FAMILY DEMOGRAPHICS	
Number of children in family	
One child	42 (18)
Two children	138 (58)
Three children	43 (18)
Four children	10 (4)
Five or more	4 (2)
Number of children with movement-related difficulties (*n* = 234)	
One	216 (92)
Two	17 (7)
Five	1 (0)
Area of province (*n* = 183)	
Vancouver Coastal Health Authority	85 (46)
Fraser Health Authority	64 (35)
Interior Health Authority	14 (8)
Northern Health Authority	3 (2)
Vancouver Island Health Authority	17 (9)
Gross family income (*n* = 174)	
Less than $20,000	4 (2)
$20,000 to less than $40,000	18 (10)
$40,000 to less than $60,000	15 (9)
$60,000 to less than $80,000	16 (9)
$80,000 to less than $100,000	31 (18)
More than $100,000	90 (52)
Family structure (*n* = 187)	
Two parents or caregivers/nuclear family in one household	152 (81)
Separated parents with dual custody	7 (4)
Stepfamily	1 (1)
Single caregiver	12 (6)
Other family structure	15 (8)
Maternal education (*n* = 187)	
Completed graduate degree	40 (21)
Bachelor degree	60 (32)
Post-secondary diploma or trades certificate	61 (33)
Some college but did not complete	13 (7)
High school diploma	11 (6)
Unknown/not applicable	2 (1)
Paternal education (*n* = 186)	
Completed graduate degree	34 (18)
Bachelor degree	50 (27)
Post-secondary diploma or trades certificate	53 (28)
Some college but did not complete	14 (8)
High school diploma	15 (8)
General educational development (GED)	2 (1)
Did not complete high school	8 (4)
Unknown/not applicable	10 (6)
Primary income earner's employment status (*n* = 186)	
Full-time	167 (90)
Part-time	13 (7)
Unemployed	5 (2)
Pension	1 (1)

### Rehabilitative Supports and Services

Table 2 outlines the rehabilitative supports and services parents have previously and/or are currently accessing. Most parents (73%; 146/199) identified that they had previously accessed therapy services to address their child's challenges; however, only 40% (56/141) reported that they are currently attending therapy services. Significant differences were noted between age of child and current access to therapy (*p* < 0.001); children who were currently being followed by a clinician were younger [median (IQR) = 9 (4.75) years] compared to those who were not accessing therapy [median (IQR) = 11 (4) years].

**Table 2:** Rehabilitative Supports and Services

Previously accessed therapy services (*n* = 142)^a^	*N* (%)
Occupational therapy	122 (86)
Physical therapy	49 (35)
Speech therapy	71 (50)
Specialized exercise program	16 (11)
Psychologist	34 (24)
Other: ABA, vision therapy, recreation therapy, counsellor, music therapy, play therapy, counsellor, Orton–Gillingham therapist, psychiatrist, CBT, therapeutic horseback riding, audiologist, ADHD coach, CYMH	29 (20)
Current therapy services (*n* = 49)^a^	
Occupational therapy	30 (61)
Physical therapy	12 (25)
Speech therapy	14 (29)
Specialized exercise program	5 (10)
Psychologist	5 (10)
Other: ABA, music therapy, recreation therapy, counsellor, play therapy, CYMH	12 (25)
Therapy services offered at school (*n* = 60)^a^	
Occupational therapy	28 (47)
Physical therapy	7 (12)
Speech therapy	26 (43)
Counseling	29 (48)
Psychology	7 (12)
Other: indigenous support, educational assistant, support for reading and writing	3 (5)
Therapy services accessed at school (*n* = 38)^a^	
Occupational therapy	19 (50)
Physical therapy	4 (11)
Speech therapy	17 (45)
Counseling	12 (32)
Psychology	1 (3)
Other: resource teacher, reading support	2 (5)

^a^ Multiple answers were permitted; summative responses do not equal 100.

ABA, applied behavioral analysis; ADHD, attention deficit hyperactivity disorder; CBT, cognitive-behavioral therapy; CYMH, Child and Youth Mental Health Program.

A small percentage of families (31%; 62/198) reported that their school offers school-based therapy services, with 63% (38/60) having accessed these services. Overall, only 20% (38/198) of children had received therapy at school.

A large number of parents (89%; 175/196) are concerned with the impact of their child's movement difficulties on their child's social and emotional health; however, only 50% (96/194) of families have accessed services and supports to address these difficulties. The most common services families accessed included counseling (48%; 43/90), psychology (19%; 17/90), and social skill classes and groups (recreational programs) (17%; 15/90). In open-ended responses, parents reported a variety of benefits, such as improved self-regulation, provision of strategies, and decreased anxiety. However, over half of parents (49/92) reported that these therapies were unsuccessful. Some parents noted in their text responses that there was a lack of understanding of DCD and the impact of motor coordination challenges on social and emotional functioning. Other parents identified time limits on these programs and lack of funding to cover the costs.

For the families who had previously or are currently accessing therapy services, 71% (41/58) felt supported to maintain progress at home; however, only 27% (38/139) felt that their child has received adequate therapy to assist with their motor coordination challenges. Contingency analyses of income and geographic location related to access to school and community-based therapy were not statistically significant (*p* > 0.05).

### School-Based Supports and Services

Prior to school entry, only 20% (41/215) of parents reported that their child had completed assessments, with 66% (21/41) noting that there were recommendations in the report. These recommendations were mostly implemented at school [70% (19/27)]. Recommendations included active therapy involvement (occupational therapy, physical therapy, and/or speech language pathology), equipment, and adaptations or modifications to classroom activities. More than three quarters of parents [78% (153/198)] identified that the classroom teacher was aware of their child's movement difficulties, with 68% (135/199) having arranged parent-teacher meetings at the beginning of the school year. Unfortunately, only 26% (50/197) reported that the teacher communicated with any treating therapists at the start of the school year. With regards to classroom supports and services, 66% (123/200) of children had an individualized education plan (IEP) and 75% (150/200) receive or previously received support from a resource teacher or educational assistant. At least three quarters of children [75% (149/200)] have classroom and/or curriculum accommodations and adaptations; however, only 57% (112/196) of children were provided with additional time to complete classroom tasks. Given that 93% (203/219) of parents report that it takes their child longer to complete movement tasks, it is disconcerting that more than 40% of children in this sample are not provided with extended time for academic activities.

With regards to physical education (PE), only 30% (36/122) of parents identified that the PE teacher communicated with them about supporting their child during gym class; however, 62% (75/121) of parents reported that they felt their child is supported to engage in PE. More concerning is that almost half of parents (59/122) indicated that their child does not feel comfortable in attending sports-related events at school.

#### Qualitative Analysis

In open-text responses, parents identified that poor teacher awareness and understanding impacted their child's successful participation at school. They also reported limited school-based supports and services to assist their child, even though close to three quarters of parents identified that teachers were aware of their child's challenges. Participant 80 commented on the lack of educator support: “Even though we have provided teachers with IEP and diagnosis, many tend to ignore or can't be bothered to help.”

Parents identified that their child's difficulty with written output significantly impacts their participation in academic activities and their ability to keep up with classroom demands. With only just over half of this sample having additional time to complete tasks, this clearly interferes with their participation and progress in the curriculum. Participant 98 identified that their child was not working to their potential: “His written output does not adequately reflect his understanding of material and therefore assessments are not reflective of his achievement potential. I am concerned for his ability to be able to write essays, exams, and learning to type.”

When parents are attempting to navigate the school systems, even with a formal diagnosis, they report a lack of supports and services, despite at least three quarters of the sample identifying that their child had classroom accommodations and received support from a resource teacher and/or educational assistant. Parents also report that even with a diagnosis of DCD, there is a lack of awareness, understanding, and clarity of eligibility criteria of whether they can qualify for a designation and an IEP. However, even with an IEP, implementation of the recommendations remains dependent on the classroom teacher, which parents identify, often lack the understanding of the scope and severity DCD can have on classroom participation. Participant 240 noted this lack of support and awareness, with limited guidance on how to ensure their child's successful participation at school:

The school seems to not have any additional resources to offer to support my child. There is limited understanding of DCD. There is limited supervision on the playground, which leads to bullying related to his physical limitations. There is no clear path how to navigate the system.

Parents identify that this lack of support often leads to social and emotional challenges, such as decreased participation, poor self-esteem, and internalizing symptomology, such as headaches, fatigue, depression, and anxiety. One parent reported their child engaged in self-harming behaviors. The SDQ (Goodman, 1997) embedded in the survey found that all children were classified in the abnormal range for emotional difficulties (100%; 98/98). Participant 75 described the emotional consequences of repeated failures, poor educator awareness, and lack of school-based supports and services:

…After years of having teacher demand he writes and journals and works with his hands, he has been exhausted mentally and I think even physically…If a kid loses the desire to learn and grow since failure has been around every corner…well explain to me how anyone could survive, never mind flourish, in a school environment.

Most parents identify a lack and/or limited availability of school-based therapy, which is consistent with the small percentage of families (31%) that identified there are therapies offered at their child's school. Even if therapies are available, parents report long waitlists and their child not meeting the eligibility criteria. Participant 55 outlined inadequate school-based services that led them to seek out alternatives:

Hire more occupational therapists and physical therapists in the school districts. One of each in a school district are not enough. My children have not been given adequate OT and PT support in the school system. We have had to seek that out through mental health services (CYMH) or through research programs such as those provided by Dr. Zwicker.

### Financial Management of Rehabilitation Supports and Service

In addition to access and availability of rehabilitative services and support, funding was also explored, to understand financial management of these services and supports. Only 39% (55/144) of families have previously received funding to cover costs of therapy services, with 45% (26/58) of families currently accessing funding. The majority of families have paid (61%) or are currently paying (55%) privately for services, with no access to additional external funding sources. In addition to monetary costs, families also identified additional costs for accessing therapy services. At least half of parents [53% (30/57)] reported that they needed to travel to therapy appointments, with a median of 45 km (range: 1–720). Almost half of parents [46% (26/57)] reported that they needed to take time off work to attend therapy appointments, with a median of 8 hr (range: 1–40) per month (*n* = 23). Children also missed school to attend therapy sessions [40% (23/57)], with a median of 3.5 hr (range: 1–16) per month (*n* = 22). Table 3 outlines the funding sources families have previously and are currently accessing, and yearly out-of-pocket expenses related to therapy costs. Families more often accessed occupational therapy through these external funding sources. Families who have previously accessed and are currently accessing funding, are still paying additional monies for therapy services and supports. Most families are paying up to $2,000 per year even when they have previously accessed (48%; 25/52) and are currently accessing (50%; 13/26) funding. For those families currently accessing therapy, median monthly cost is $240 (range: 0–2650) (*n* = 38). The majority of families are using funds from the provincial Autism program; however, eligibility is dependent on a co-occurring diagnosis of ASD. Despite access to provincial funding, these families report that they are still paying up to $2,000 per year (42%; 11/26) in additional therapy costs, with some families reporting paying upwards of $4,000 per year (23%; 6/26) and $6,000 per year (12%; 3/26).

**Table 3:** Funding for Rehabilitative Services and Supports

Previously accessed funding source (*n* = 44)^a^	*N* (%)
Autism funding	20 (46)
Variety Children's Charity	10 (23)
Extended health insurance	7 (16)
Homeschool/distance education learning	4 (9)
CKNW	3 (7)
Government agencies and organizations (e.g., Ministry of Child and Family Development)	16 (36)
Other: family and bursary from independent school	3 (7)
Current funding source (*n* = 24)^a^	
Autism funding	14 (58)
Variety Children's Charity	4 (17)
Extended health insurance	2 (8)
Home school funding	3 (13)
Government agencies/organizations	4 (17)
Other: bursary from independent school	1 (4)
Yearly therapy costs (*n* = 142)	
None	11 (21)
$1–1,999	25 (48)
$2,000–3,999	10 (19)
$4,000–5,999	2 (4)
$6,000–7,999	2 (4)
$8,000–9,999	1 (2)
$10,000 and up	1 (2)

^a^ Multiple answers were permitted; summative responses do not equal 100.

#### Qualitative Analysis

Parents report the need for school-based supports and services, as funding and finances are both barriers and facilitators for rehabilitative services and supports. Those with funding and financial means can likely access rehabilitative services and supports that are not provided through the school. Parents reported limited access to school-based assessments and therapy can lead them to seek out private testing and rehabilitation services to promote their child's development. Participant 78 paid for private therapy services; however, the lack of in-school services limited educator access to therapy strategies and recommendations:

We are lucky to be able to afford sending our child to an OT; however, for many families I am sure budget and work schedules make it difficult…My child is still on the waitlist for a school OT which we know he will never see. That resource is also not available for the teaching staff to consult. This means all experts are brought into the school by us and paid for by us which we are happy to do but limits the amount of time that the teachers are in contact with experts…and it shows.

### Parent-Identified Needs and Resources

In open-ended responses, parents identified that resource allocation to better support children and families impacted by movement difficulties needs to focus on increasing access, availability, and funding of school-based and rehabilitative supports and services, as well as increase resources for families, educators, and community programmers. Parents identified the need for increased awareness of the etiology, symptomology, and impact of DCD for educators, health professionals, and people in the community (parents, government, and other community members).

Parents identified that their areas of highest priority were ensuring that their child is successful and supported at school through: (1) availability and access to school-based services; (2) resources; (3)accommodations and modifications to academic expectations; (4) access to educational assistants and equipment; and (5) increased capacity amongst educators on the impact of DCD on school function. Parents reported the need for increased availability and access to rehabilitative services, specifically, occupational therapy. Services and support for social and emotional health and funding to cover assessments, treatments, and resources were also areas of parent-identified needs.

Parents acknowledged inconsistent access to a timely diagnosis, essential for early detection and intervention. Services for social and emotional health became a greater priority as their child aged. For those families with children ages 13 and up, the need for supports and services for social and emotional health are greater, as compared to supports and services for motor skill development.

## Discussion

### Access and Availability of Rehabilitative and School-Based Services and Supports

Disconcertingly, families appear to be struggling to access supports and services, critical to meet their child's needs at school and in the community. These results are consistent with parental experiences when attempting to procure therapeutic services and supports in Ontario (Missiuna et al., 2006; Rodger & Mandich, 2005) and other countries (Licari et al. 2021; Maciver et al., 2011; Novak et al., 2012). Only two out of five children are currently accessing therapy to support their movement challenges. The majority of these families are accessing these services through the private sector with limited options for funding. Active participation in therapy appears to reduce with age, despite persistent movement challenges. Parents of older children in this study prioritize services and supports for mental health compared to services for physical skills. This is consistent within the literature on the cumulative impacts of early motor experiences leading to mental health challenges through to adulthood (Blank et al., 2019; Missiuna et al., 2007; Tal-Saban & Kirby, 2018). This pattern of therapy usage is similar in the UK; parents reported motor goals for younger children and mental health goals for older children (Cleaton et al., 2020). However, parents in our study reported low satisfaction with mental health services. It may be that mental health professionals are using traditional approaches for treating anxiety and depression as primary diagnoses, rather than addressing these symptoms as secondary to DCD. Families in British Columbia and internationally (Licari et al., 2021; Maciver et al., 2011; Novak et al., 2012; Rodger & Mandich, 2005) identified a lack of understanding amongst health-care providers on the impact of DCD on daily function, contributing to limited efficacy in care models. Decline in service use may also be due to difficulty in accessing funded programs and services and/or the financial cost. The financial burden of private therapy may increase as their child ages. Demographically, children age 8 to 12 years were overrepresented, which may also elucidate this decreased therapy use, as priorities change and financial burden increases.

Inconsistent access to school-based therapy services may have led parents to seek community-based therapy, with most accessing occupational therapy. Access to occupational therapy is only covered under provincial healthcare funding in hospitals and rehabilitation centers (Canadian Association of Occupational Therapists, 2016). The lack of access and availability of school-based therapy services can lead to inequitable access, as those families with financial means and funding can choose to pay for private therapy.

It would appear that inconsistent access to school-based resources, limited active therapy involvement, and ineffective services for social and emotional well-being may lead to higher risks of emotional and social challenges, particularly as all children in this sample met the criteria for abnormal emotional function and peer relationships on the SDQ (Goodman, 1997). It is therefore a critical need in British Columbia for funded and accessible multi-disciplinary school and community-based supports and services for children with DCD.

### Access and Availability of Funding

There is no standard of care and dedicated funding in British Columbia for children with DCD, which is reflective that majority of parents in this sample are paying for therapy, in addition to monetary costs of travel time, absences from work, and child absences from school. Access to government funding appears to be limited to those families who qualify for provincial autism funding; however, even these families incur additional therapy costs. The high cost of care for DCD with limited funding options is a stark reality for many families (Cleaton et al., 2020). Access to other funding sources, such as Variety Children's Charity and CKNW, typically have application criteria based on diagnosis and income, which limit access and many families might not be aware of these sources. Extended benefits do not consistently cover all therapies, particularly occupational therapy. An outcome-based standard of care with dedicated funding for children with DCD is essential, to ensure equitable access to therapy services. This will ensure that all children with DCD and their families have the same opportunity to benefit from care.

### Clinical Implications for Policy Development

Parents report the lack of supports and services, funding, and understanding of DCD have a detrimental effect on themselves and the family dynamic. To affect change for children with DCD and their families, there needs to be a multi-disciplinary approach, where all facets of function are addressed. Families are mainly accessing occupational therapy services; however, there is clear gap in the need for services and support to address social and emotional impacts of DCD. Therapy and school supports are integral environmental supports to build and develop a child's social and personal resources, which has been outlined as key mitigating factors in developing internalizing symptomology (Cairney et al., 2013; Mancini et al., 2016). The environmental stress hypothesis posits that continued exposure to secondary environmental stressors, such as peer conflict and victimization resulting from early motor coordination challenges and decreased engagement and participation, increase the risks for developing internalizing symptomology (Cairney et al., 2013; Mancini et al., 2016). As occupational therapists, we need to advocate for holistic evidence-based service approaches and ensure that we are providing best practice that does not just focus on the motor challenges seen with DCD.

Overwhelmingly, regardless of the age of their child, parents identify as their highest priority is the need for school-based supports and services. Increased funding and availability of school-based services would lessen the need for parents to seek out private therapy, which for many is unattainable. Partnering for Change (P4C) is an example of a tiered service delivery model, which aims to build educator capacity through a collaborative partnership between the teacher and therapist (Missiuna et al., 2012). Through use of this model, occupational therapists were able to identify children at risk for DCD at a younger age and those that likely would not have been referred for occupational therapy services, with a potential to eliminate waitlists for services (Missiuna et al., 2017). Building educator capacity and school-based supports would promote early detection and accessibility of services (Camden et al., 2015; Missiuna et al., 2017), leading to lower risks of the chronic, cumulative and long-term physical, social, emotional and psychological impacts associated with DCD. Children with DCD can achieve functional improvements with school-based therapy services, as compared to clinic-based services (Ward et al., 2017). These results reiterate the need for a standard of care that incorporates increased access and availability of school-based services and supports, including access to school-based therapy for children with DCD in British Columbia The current system does not appear to be meeting parent and child needs, likely leading to increased risks of emotional and psychological sequelae.

As occupational therapists, we need to take an active role to advocate for services and supports for children with DCD. The DCD Advocacy Toolkit (Montgomery et al., 2018) outlines the essential steps therapists can do to change the narrative for children and their families with DCD. There is no coordinated service delivery with proper funding in British Columbia to assist families with navigating the healthcare and school systems for rehabilitative supports and services. A pattern of inequitable service delivery emerges, with those families having the financial means, funding, and resources to access services and supports.

Through this research, we aim to develop a provincial policy brief, to advocate for changes and develop a standard of care in British Columbia. As a profession, we need to increase our understanding and awareness of the global impact DCD has on children and their families and use our voice to impart and empower families and ourselves, to change the narrative.

### Limitations

This study is not without limitations. Demographically, this sample reflects a high socio-economic status from urban areas in the province, with a median gross income > $100,000 annually and primarily university educated parents/caregivers. Most children come from two-parent households with the primary income earner working full-time. There was limited representation from families in rural and/or remote locations with varying levels of socio-economic status, impacting generalizability and scope of findings. Results may have differed if there was broader representation across the province.

Families from higher socio-economic backgrounds tend to have greater availability of resources, enabling them to pay for additional services and supports, possibly skewing the results. However, families in this subset have identified significant barriers regarding access, funding, and resources, impacting their ability to navigate the healthcare and school systems. If these families are struggling, it is likely that the issues are magnified for families with less education or financial resources. Participant age tended to be overrepresented in the 8 to 12 years age band, which also may bias results.

An inherent bias also exists for those that tend to volunteer to participate in research studies, which may under-represent the true needs of families of children with DCD. There was limited accessibility of the questionnaire, as it was distributed through an online platform in English; as such, parents required email access and grasp of the English language, which may also impact the generalizability of the results. To reflect study aims, not all questions from the online survey were analyzed. Future papers could address other categories in the questionnaire, such as activity and participation patterns in the community, for a deeper understanding of the global impact of DCD on child and family function.

Future research aims to distribute the impACT for DCD survey across Canada, for a comparative analysis on how parents navigate the healthcare and school systems for rehabilitative supports and services, and to promote national awareness and advocacy for children with DCD and their families. National dissemination of this survey will provide a better understanding of successes and gaps of varied provincial frameworks of service delivery, to inform national standards of care and best practice. This research will guide the development of pan-Canadian standards of care for policy and decision-making, ensuring families of children with DCD receive supports and services, essential for their child's development.

## Conclusion

Families with children with DCD struggle to access rehabilitative services and supports at school and in the community. The current system in British Columbia is failing to meet parent-identified needs, to ensure their child is able to develop and participate at home, at school and in the community. Parents report secondary mental health consequences associated with DCD, with at least half of the children having co-occurring anxiety and all children scoring in the abnormal range for emotional difficulties. There needs to be systemic changes to service delivery for children with DCD, starting with access and availability of school-based services and supports, in addition to funding for community-based therapy services. Increased awareness and understanding of DCD for educators, health professionals, and people in the community are essential for early detection, intervention, and advocacy for services and supports. Dedicated funding for children with DCD is essential to ensure equitable access of services. A coordinated and funded service delivery will ensure families can be successful to provide their children with opportunities to flourish and participate while minimizing the chronic and negative secondary consequences associated with DCD. As occupational therapists, we are ideally suited to address both the physical and mental health needs of children with DCD and we need to advocate for systemic change to better support children with DCD and their families.

### Key Messages

- A standard of care needs to be developed specific for occupational therapy assessment and intervention for children with developmental coordination disorder (DCD).
- Occupational therapists need to advocate for funding for school-based and community therapy to better support children with DCD and their families.
- Standardized pathways are needed for coordinated services and supports at school to help children with DCD meet their academic potential and positively contribute to their well-being.

## Supplemental Material

sj-docx-1-cjo-10.1177_00084174231197618 - Supplemental material for Success or Failure? Are We Meeting the Needs of Children With Developmental Coordination Disorder? Supplemental material, sj-docx-1-cjo-10.1177_00084174231197618 for Success or Failure? Are We Meeting the Needs of Children With Developmental Coordination Disorder? by Erin S. Klein, Melissa Licari, Skye Barbic and Jill G. Zwicker in Canadian Journal of Occupational Therapy
