# Supplementary material for: Success or Failure? Are We Meeting the Needs of Children With Developmental Coordination Disorder?
Source: Can J Occup Ther. 2023 Sep 6;91(2):149–59. doi: 10.1177/00084174231197618 (PMC11088219; doi:10.1177/00084174231197618)
Supplement: sj-docx-1-cjo-10.1177_00084174231197618 - Supplemental material for Success or Failure? Are We Meeting the Needs of Children With Developmental Coordination Disorder? [file sj-docx-1-cjo-10.1177_00084174231197618.docx]

**Appendix**

Impact for DCD Questionnaire


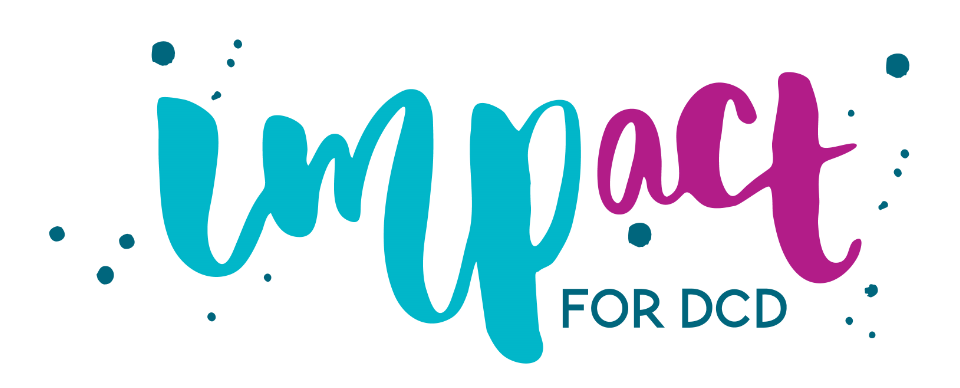


Learning to walk, talk, eat, run and play are important skills we develop during childhood. Unfortunately, for some children, they are not always easily learned. When a child experiences difficulties in their movement skills and these challenges continue over a prolonged period of time, they are often referred to as having motor planning difficulties and sometimes a condition medically diagnosed as Developmental Coordination Disorder (DCD).

As a parent or guardian of a child impacted by prolonged movement difficulties, you have first-hand experience of the many challenges, including access to therapy, educational support, along with the physical, mental and financial impacts on your child and family. Unfortunately, awareness of these issues is currently limited in the medical, education, and government systems in British Columbia. This needs to change and to do this, we need your help.

We invite you to participate in this survey if you have a child/ren aged 5-18 years who has persistent movement difficulties not associated with another movement-related condition (e.g., cerebral palsy).

Building on a similar survey conducted in Australia by Dr. Melissa Licari and her team, this project is led by researchers at the University of British Columbia, BC Children’s Hospital, and Sunny Hill Children’s Centre for Children. We will collect information to show the impact of movement-related difficulties known as DCD. In doing so, we hope to increase awareness, initiate action to provide better support services to families, schools and medical centres, and to bring about change in how children with DCD receive support and services in our province.

The survey has a series of questions to answer, and should take ~30 minutes to complete. Participation in this survey is entirely voluntary. In completing the survey, your identity will remain anonymous.

The survey might ask questions about topics that at times you may find sensitive. But this information is very important for us to truly understand your experience. If at any point you do not wish to continue with the survey, you can stop immediately.

We kindly ask that you to take your time responding to the questions, and to please forward this questionnaire on to any other families you know impacted by similar movement-related difficulties. Thank you in advance for your participation.

By checking this box, you are indicating your consent to participate in this study. You can withdraw your consent at any time during the process of completing this questionnaire.

Details of the survey’s investigators are included below, should you wish to contact them concerning any parts of the survey:

Erin Klein, Occupational Therapist

Master’s Student in Rehabilitation Sciences

University of British Columbia

[erin.klein@alumni.ubc.ca](mailto:erin.klein@alumni.ubc.ca)

Dr. Jill Zwicker

Associate Professor | Department of Occupational Science & Occupational Therapy

Associate Member | Department of Pediatrics (Division of Developmental Pediatrics)

Faculty of Medicine | The University of British Columbia| Vancouver

Investigator | BC Children’s Hospital Research Institute

Clinician Scientist | Sunny Hill Health Centre for Children

Associate Investigator | Kids Brain Health Network

Associate Member | CanChild Centre for Childhood Disability Research

jill.zwicker@ubc.ca

 
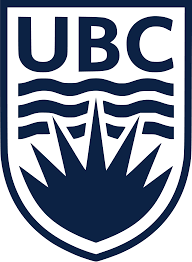
    **
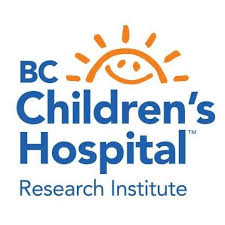
**
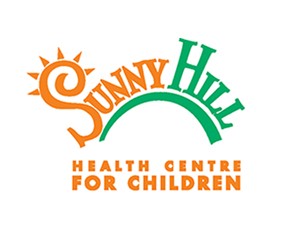


Start of Block: Family and Diagnostic Information

How many children do you have?

- 1 (1)
- 2 (2)
- 3 (3)
- 4 (4)
- 5+ (5)

How many of your children have movement-related difficulties?

________________________________________________________________

Is there any history of movement-related difficulties in your family?

- yes (1)
- no (2)

| Page Break |  |
| --- | --- |

Display This Question:

If Is there any history of movement-related difficulties in your family? = Yes

Who else in your family has movement difficulties and what difficulties do they have? Please include the relationship of the family member (e.g. father, mother, uncle, etc.) and not their name or other personally identifiable information

________________________________________________________________

________________________________________________________________

________________________________________________________________

________________________________________________________________

________________________________________________________________

Is there any history of any other medical condition affecting movement in your family?

- Yes (1)
- No (2)

Display This Question:

If Is there any history of any other medical condition in your family? = Yes

If there is a history of any other medical condition affecting movement in your family, please check those that apply

- attention deficit hyperactivity disorder (1)
- autism spectrum disorder (2)
- dyslexia (3)
- learning difficulties (4)
- intellectual impairment (5)
- global developmental delay (6)
- tic disorder (7)
- Other (please specify) (8) ________________________________________________

Display this question:

If there are a history of movement-related difficulties in your family= yes:

Would you be willing to be contacted to find out more about a genetic study for DCD? If yes, please provide your name and email address. Your survey responses to this questionnaire would remain confidential and de-identified, and your contact information would not be linked to the survey responses. Providing your name and email would only be used to send **information** about the genetics study. A separate consent process would be required to participate in the genetics study, if you provide us with your contact information.

Name: _________________________________

Email Address: ___________________________

Given that the impact is likely to vary from child to child, we kindly ask that you firstly complete the survey for your eldest child with movement-related difficulty. On completion, the questions will reload for each subsequent child with a movement-related difficulty

End of Block: Family and Diagnostic Information

Start of Block: Movement difficulties

**Please answer the following questions for ${lm://Field/1}${lm://Field/2} Child with movement difficulties**

Child's current age in years?

________________________________________________________________

What is the child's sex?

- Male (1)
- Female (2)

What is child's position in family?

- Eldest (1)
- Middle (2)
- Youngest (3)
- Only child (4)
- Other, please specify (5)

______________________________________

S

- yes (1)
- no (2)

Gestational age in weeks:

_________________________________________________________________

How old was your child in years when you first became concerned with their movement?

What environment were these issues first observed or raised?

- home (1)
- mothers/parents group (2)
- play group (3)
- child care (4)
- kindergarten (5)
- school (6)
- public health nurse (7)
- infant development consultant (8)
- supported child development consultant (9)
- Other (please specify) (10) ________________________________________________

How old was your child when you first sought help?

________________________________________________________________

Has your child been given a diagnosis for their movement difficulties?

- yes (1)
- no (2)

Skip To: Q17 If Has your child been given a diagnosis for their movement difficulties? = yes

Has your child’s movement difficulties been described as any of the following? (please check all that apply)

- motor planning difficulties (1)
- motor coordination problems (2)
- perceptual-motor dysfunction (3)
- sensory integration difficulties (4)
- sensorimotor dysfunction (5)
- developmental coordination disorder (6)
- other (please specify) (7) ________________________________________________

Who provided you with this description? (please check all that apply)

- General Practitioner/family doctor(1)
- Community Paediatrician (2)
- Developmental paediatrician (3)
- Other medical specialist; such as neurologist, neonatologist, sports medicine doctor, psychiatrist, etc. (4)
- Occupational therapist (5)
- Physiotherapist (6)
- Psychologist (7)
- other (please specify) (8) ________________________________________________

How old was your child when they were described in this way?

________________________________________________________________

Where did you turn for information and support?

________________________________________________________________

________________________________________________________________

________________________________________________________________

________________________________________________________________

________________________________________________________________

What is/has been your main source of information?

________________________________________________________________

________________________________________________________________

________________________________________________________________

________________________________________________________________

If you are in the process of trying to get a diagnosis for your child, please describe this process.

_____________________________________________________________________

_____________________________________________________________________

_____________________________________________________________________

|  |
| --- |

Has your child been diagnosed with any of the following conditions? (please check all those that apply)

- developmental coordination disorder (DCD) (1)
- dyspraxia (2)
- motor dyspraxia (3)
- developmental dyspraxia (4)
- ideomotor dyspraxia (5)
- ideational dyspraxia (6)
- oromotor dyspraxia (7)
- constructional dyspraxia (8)
- sensory integration disorder (9)
- deficit in attention and motor perception (DAMP) (10)
- hypermobility (11)
- Other (please specify) (12) ________________________________________________

Who provided you with this diagnosis? (please check all those that apply)

- General practitioner/family doctor (1)
- Community paediatrician (2)
- Developmental paediatrician (3)
- Other medical specialist; such as neurologist, neonatologist, sports medicine doctor, psychiatrist, etc. (4)
- Occupational Therapist (5)
- Physiotherapist (6)
- Psychologist (7)
- Other (please specify) (8)

What information helped inform the diagnosis? (please check all that apply)

- teacher report (1)
- clinician report (occupational therapist, physiotherapist, psychologist) (2)
- parent report (3)
- formal assessment (4)
- other (5)

How old was your child when they were diagnosed?

________________________________________________________________

What was the length of time from identifying a concern with your child’s movement to a formal diagnosis:

- - Less than 6 months (1)
  - 6-12 months (2)
  - 1-2 years (3)
  - 3-4 years (4)
  - More than 4 years (5)

Was there a financial cost associated with determining a diagnosis?

- yes (1)
- no (2)

If yes, please specify amount:

Had you heard of the disorder before diagnosis?

- yes (1)
- no (2)

Was receiving this diagnosis helpful?

- yes (1)
- no (2)

Why was receiving this diagnosis helpful or not helpful?

________________________________________________________________

________________________________________________________________

________________________________________________________________

________________________________________________________________

________________________________________________________________

Is there anything else you would like to add about the process about obtaining a diagnosis for your child?

__________________________________________________________________

__________________________________________________________________

__________________________________________________________________

__________________________________________________________________

Where did you turn for information and support?

________________________________________________________________

________________________________________________________________

________________________________________________________________

________________________________________________________________

________________________________________________________________

What is/has been your main source of information?

________________________________________________________________

________________________________________________________________

________________________________________________________________

________________________________________________________________

________________________________________________________________

Does your child have any co-occurring conditions? (please choose all that apply)

- Autism Spectrum Disorder (1)
- ADHD (2)
- learning difficulties (3)
- apraxia (4)
- childhood apraxia of speech/verbal dyspraxia/specific language impairment/speech difficulties (5)
- dysgraphia (6)
- dyslexia (7)
- epilepsy (8)
- anxiety (9)
- depression (10)
- Other (please specify) (11) ________________________________________________

End of Block: Movement difficulties

Start of Block: Activity

**Please answer the following questions for ${lm://Field/1}${lm://Field/2} Child with movement difficulties**

What terms best describe your child’s movement at present (please check all that apply)?

- uncoordinated (1)
- awkward (2)
- disorganized (3)
- poorly timed (4)
- clumsy (5)
- unstable (6)
- slow (7)
- rushed (8)
- heavy (9)

What activities does your child have difficulty completing (please check all that apply)?

- dressing (e.g., buttons, zips, laces) (1)
- eating (e.g., using utensils, pouring a drink) (2)
- self-care (e.g., brushing hair, cleaning teeth) (3)
- helping with household chores (4)
- toileting (5)
- drawing/writing (6)
- cutting with scissors (7)
- craft activities (8)
- reading (9)
- playing lego/construction/puzzles (10)
- playing board games/cards (11)
- active outside play (12)
- bike riding/scooting (13)
- trampolining (14)
- ball skills - throwing, catching, kicking (15)
- climbing a tree (16)
- skipping (17)
- swimming (18)
- sledding (19)
- skiing/snowboarding (20)
- skating (21)
- playing on the playground (22)
- Other (please specify) (23) ________________________________________________

|  | yes (1) | no (2) |
| --- | --- | --- |
| Do you think it takes your child longer than other children to accomplish movement tasks? (2) |  |  |
| Does your child become fatigued performing movement tasks they find difficult (3) |  |  |
| Compared to other children, do you think your child is more tired at the end of the day? (4) |  |  |

On average, how many hours of sleep does your child have per night?

________________________________________________________________

If your child was given 30 minutes to themselves at home, what would they choose to do?

________________________________________________________________

________________________________________________________________

________________________________________________________________

________________________________________________________________

________________________________________________________________

How many minutes per day on average does your child spend watching television or engaging in electronic or screen-based activities?

________________________________________________________________

Does your child enjoy participating in organized sport and physical activity

- yes (1)
- no (2)

Why or why not?

| Page Break |  |
| --- | --- |

At present, does your child participate in any organized sport, physical or leisure activities?

- yes (1)
- no (2)

Display This Question:

If At present, does your child participate in any organized sport, physical or leisure activities? = yes

Please indicate the name of each organized sport, physical or leisure activity in which your child is currently engaged, and the length of time spent doing the activity in minutes per week

|  | Minutes per week |
| --- | --- |
|  | (1) |
| Activity 1 (1) |  |
| Activity 2 (2) |  |
| Activity 3 (3) |  |
| Activity 4 (4) |  |
| Activity 5 (5) |  |

Do you think your child engages in at least 60 minutes of moderate to vigorous physical activity per day?

- yes (1)
- no (2)

Skip To: Q41 If Do you think your child engages in at least 60 minutes of moderate to vigorous physical activity... = yes

How many minutes of moderate to vigorous activity do you think they engage in per day?

________________________________________________________________

Are you concerned about the impact your child’s movement difficulties have on their physical health?

- yes (1)
- no (2)

Display This Question:

If Are you concerned about the impact your child’s movement difficulties have on their physical heal... = yes

What is your biggest concern?

________________________________________________________________

________________________________________________________________

________________________________________________________________

________________________________________________________________

________________________________________________________________

End of Block: Activity

Start of Block: Schooling

**Please answer the following questions for ${lm://Field/1}${lm://Field/2} Child with movement difficulties**

Did your child complete any evaluations on their entry into the school system?

- yes (1)
- no (2)

Skip To: Q46 If Did your child complete any evaluations on their entry into the school system? = no

Did they report any findings related to your child’s movement difficulties?

- yes (1)
- no (2)

Display This Question:

If Did they report any findings related to your child’s movement difficulties? = yes

What recommendations were made?

________________________________________________________________

________________________________________________________________

________________________________________________________________

________________________________________________________________

________________________________________________________________

Based on the following scale, to what degree are these recommendations being implemented?

| Always (1) | Very often (2) | Sometimes (3) | Rarely (4) | Never (5) |
| --- | --- | --- | --- | --- |

Current grade/year level

________________________________________________________________

Does your child attend a public, private, independent school or is home schooled?

- public (1)
- private (2)
- independent (3)
- home schooled (4)

Display this question if checked home school:

What are the main reasons you have chosen to home school your child?

Skip to question 73 if does your child attend a public, private, independent school or is home schooled, home school= yes

At the beginning of the current school year

|  | Yes (1) | No (2) |
| --- | --- | --- |
| Was your classroom teacher aware of your child’s movement difficulties? (1) |  |  |
| Did they meet with you to discuss your child’s needs? (2) |  |  |
| Did they engage/communicate with any therapists your child was seeing? (3) |  |  |
| Did they put an individual education plan (IEP) in place? (4) |  |  |

Has your child ever received support from resource teacher or educational assistant to assist with tasks impacted by their movement difficulties (e.g., handwriting)?

- yes (1)
- no (2)

Skip To: Q54 If Has your child ever received support from a teacher/integration/education aid to assist with task... = no

Are there classroom or curriculum accommodations or adaptations for your child?

- Yes (1)
- No (2)

Skip To: Q54 If Does the school provide provisions/spaces has to support your child’s learning? = no

What are the classroom accommodations or adaptations that have been or are currently being provided?

________________________________________________________________

________________________________________________________________

________________________________________________________________

________________________________________________________________

________________________________________________________________

Is your child provided with additional time to complete tasks that are impacted by their movement difficulties (e.g., tests/exams)?

- yes (1)
- no (2)

Is your child tired at the end of a school day?

- yes (1)
- no (2)

Does your child’s school have a physical education teacher?

- yes (1)
- no (2)

Skip To: Q58 If Does your child’s school have a physical education teacher? = no

If your child has a physical education teacher,

|  | yes (1) | no (2) |
| --- | --- | --- |
| Has the physical education teacher ever communicated with you or the classroom teacher about supporting your child? (2) |  |  |
| Do you feel your child is supported to engage in physical education classes at school? (3) |  |  |
| Does your child feel comfortable attending sport- themed school events, such as sports day, intramurals and/or sport-themed assemblies (4) |  |  |

Are therapy services offered within the school?

If yes:

Please check all that apply:

- Occupational therapy (1)
- Physical therapy (2)
- Speech Language therapy (3)
- Psychologist (4)
- Counselling (5)
- Other: (6)

please describe: ______________________________________

Who does your child see and how often/frequency?

What are the major challenges for your child at school?

________________________________________________________________

________________________________________________________________

________________________________________________________________

________________________________________________________________

________________________________________________________________

Does your child enjoy going to school?

- yes (1)
- no (2)

Has your child had difficulty making friends at school?

- yes (1)
- no (2)

Do you think your child’s movement difficulties are negatively impacting their ability to reach their potential at school?

- yes (1)
- no (2)

Do you have concerns about how your child’s movement difficulties may impact their ability to successfully complete their schooling to the best of their potential?

- yes (1)
- no (2)

Skip To: Q65 If Do you have concerns about how your child’s movement difficulties may impact on their ability to... = no

What is your biggest concern on how your child’s movement difficulties may impact their ability to successfully complete their schooling to the best of their potential?

________________________________________________________________

________________________________________________________________

________________________________________________________________

________________________________________________________________

________________________________________________________________

Do you have concerns about your child’s movement difficulties affecting their ability to gain employment in the future?

- yes (1)
- no (2)

End of Block: Schooling

Start of Block: Therapy/Intervention

**Please answer the following questions for ${lm://Field/1}${lm://Field/2} Child with movement difficulties**

Have you ever accessed therapy to assist your child with their movement difficulties?

- yes (1)
- no (2)

Display This Question:

If Have you ever accessed therapy to assist your child with their movement difficulties? = no

Please state your reasons why you haven't accessed therapy

________________________________________________________________

________________________________________________________________

________________________________________________________________

________________________________________________________________

________________________________________________________________

Display This Question:

If Have you ever accessed therapy to assist your child with their movement difficulties? = yes

What therapy services have you accessed and for how long? e.g., accessing therapy for 18 months would be 1 year, 6 months

|  | How long? | |
| --- | --- | --- |
|  | Years (1) | Months (2) |
| Occupational therapy (1) |  |  |
| Physical therapy (2) |  |  |
| Speech therapy (3) |  |  |
| Specialized exercise programs (4) |  |  |
| Psychologist (5) |  |  |
| Other – specify (6) |  |  |

Are you currently attending any therapy services to assist their child with their movement difficulties?

- yes (1)
- no (2)

Skip To: Q78 If Are you currently attending any therapy services to assist their child with their movement diffic... = no

What therapy services are you currently accessing to assist your child with their movement difficulties? How often do you access these services, and what is the estimated out of pocket monthly expense for these services?

|  | How often? | Monthly out of pocket expense |
| --- | --- | --- |
|  |  | Other (1) |
| Occupational therapy (1) | ▼ Daily (1) ... Other (6) |  |
| Physical therapy (2) | ▼ Daily (1) ... Other (6) |  |
| Speech therapy (3) | ▼ Daily (1) ... Other (6) |  |
| Specialized exercise programs (4) | ▼ Daily (1) ... Other (6) |  |
| Psychologist (5) | ▼ Daily (1) ... Other (6) |  |
| Other – specify (6) | ▼ Daily (1) ... Other (6) |  |

Do you currently receive any funding to support your child’s therapy costs?

- yes (1)
- no (2)

Display This Question:

If Do you currently receive any funding to support your child’s therapy costs ? = yes

Please list providers and what therapy it supports?

|  | Therapy type |
| --- | --- |
|  | Answer 1 (1) |
| Provider 1 (1) |  |
| Provider 2 (2) |  |
| Provider 3 (3) |  |

Do you need to travel to attend therapy sessions?

- yes (1)
- no (2)

Display This Question:

If Do you need to travel to attend therapy sessions? = yes

On average, how far would you travel each week or each month to access therapy services? (km)

________________________________________________________________

Do you have to take time off work to enable your child to attend therapy sessions?

- yes (1)
- no (2)

Display This Question:

If Do you have to take time off work to enable your child to attend therapy sessions? = yes

How many hours per month do you need to take off work for therapy appointments for your child?

________________________________________________________________

If your child is school-aged, do they miss any of their school classes to attend therapy sessions?

- yes (1)
- no (2)

Display This Question:

If If your child is school-aged, do they miss any of their school classes to attend therapy sessions? = yes

How many minutes per week do they miss of school?

________________________________________________________________

Have you felt supported to maintain the progress that your child makes during their therapy time at home?

- yes (1)
- no (2)

Do you feel your child has received sufficient therapy to assist their movement difficulties?

- yes (1)
- no (2)

What do you think your average yearly out of pocket expense have been accessing movement related-therapy for you child?

- None(1)
- $1-$1999
- $2000-$3999 (2)
- $4000-$5999 (3)
- $6000-$7999 (4)
- $8000-$9,999 (5)
- $10,000+> (6)

Has your child ever received any funding to support their therapy costs?

- yes (1)
- no (2)

Display This Question:

If Has your child ever received any funding to support their therapy costs? = yes

Please list providers and what therapy did it support?

|  | Therapy type |
| --- | --- |
|  | Answer 1 (1) |
| Provider 1 (1) |  |
| Provider 2 (2) |  |
| Provider 3 (3) |  |

End of Block: Therapy/Intervention

Start of Block: Social & Emotional Impact on the Child

**Please answer the following questions for ${lm://Field/1}${lm://Field/2} Child with movement difficulties**

Are you concerned about the impact your child’s movement difficulties have on their social and emotional health?

- yes (1)
- no (2)

Display This Question:

If Are you concerned about the impact your child’s movement difficulties have on their social and em... = yes

What are your biggest concerns?

________________________________________________________________

________________________________________________________________

________________________________________________________________

________________________________________________________________

________________________________________________________________

Did you access supports to help with your child’s social and emotional health?

- yes (1)
- no (2)

Please describe the type of supports:

Did you find that the programs assisted your child in dealing with the impact of their movement difficulties on their social and emotional health?

- yes (1)
- no (2)

| Please describe: Please answer the following about your child | Always (1) | Very often (2) | Sometimes (3) | Rarely (4) | Never (5) |
| --- | --- | --- | --- | --- | --- |
| Does your child withdraw or avoid participating in movement-related activity? (1) |  |  |  |  |  |
| Is your child anxious learning or performing movement-related activities? (2) |  |  |  |  |  |
| Does your child experience difficulty socializing with peers? (3) |  |  |  |  |  |
| Does your child have difficulty making friends? (4) |  |  |  |  |  |
| When communicating, does your child make eye contact and orient their body appropriately? (5) |  |  |  |  |  |
| Does your child talk at the right time in a conversation? (6) |  |  |  |  |  |
| Does your child keep still and listen when another person is talking? (7) |  |  |  |  |  |
| How often does your child feel happy? (8) |  |  |  |  |  |
| How often does your child feel sad? (9) |  |  |  |  |  |
| How often does your child feel angry or made? (10) |  |  |  |  |  |

In the past 6 months, has your child:

|  | Very true (1) | Somewhat true (2) | Not true (3) |
| --- | --- | --- | --- |
| Often complained of headaches, stomach aches, or sickness? (1) |  |  |  |
| Had many worries or often seem worried? (2) |  |  |  |
| Seemed unhappy, down-hearted, or tearful? (3) |  |  |  |
| Seemed nervous or clingy in new situations? (4) |  |  |  |
| Had many fears or been easily scared? (5) |  |  |  |
| Seemed solitary or tended to play alone? (6) |  |  |  |
| Had at least one good friend? (7) |  |  |  |
| Been generally liked by other children? (8) |  |  |  |
| Been picked on or bullied by other children? (9) |  |  |  |
| Gotten along better with adults than with children? (10) |  |  |  |
| Been considerate of other people’s feelings? (11) |  |  |  |
| Shared readily with other children (e.g., treats, toys, pencils)? (12) |  |  |  |
| Been kind to younger children? (13) |  |  |  |
| Volunteered to help others (e.g., parents, teachers, other children)? (14) |  |  |  |

How often does your child’s movement difficulties cause the following impact on the family?

|  | Always (1) | Very often (2) | Sometimes (3) | Rarely (4) | Never (5) |
| --- | --- | --- | --- | --- | --- |
| Emotional worry or concern? (1) |  |  |  |  |  |
| Limit the amount of time you have for your own personal needs? (2) |  |  |  |  |  |
| Limit the types of activities you are able to do as a family? (3) |  |  |  |  |  |
| Financial strain? (4) |  |  |  |  |  |
| Concern about their future? (5) |  |  |  |  |  |

How would you rate your physical health, in relation to having a child with motor coordination difficulties:

- 1 Excellent (1)
- 2 Very good (2)
- 3 Good (3)
- 4 Fair (4)
- 5 Poor (5)

How rate your mental health, in relation to having a child with motor coordination difficulties:

- 1 Excellent (1)
- 2 Very good (2)
- 3 Good (3)
- 4 Fair (4)
- 5 Poor (5)

Please describe any further detail as to your physical health and mental health, in relation to having a child with motor coordination difficulties.

______________________________________________________________________________________________________________________________________________________________________________________________________________________________________________________________________________________________________________________________________________________________________________________________________

End of Block: Social & Emotional Impact on the Child

Start of Block: Demographic Information

In which area of the province do you live in?

- Interior (1)
- Fraser (2)
- Vancouver Coastal (3)
- Vancouver Island (4)
- Northern (5)

What are the first three digits of your postal code?

________________________________________________________________

What is your gross family income ?

- Less than $20,000 (1)
- $ 20,000 to less than $40,000 (2)
- $40,000 to less than $60,000 (3)
- $60,000 to less than $80,000(4)
- $80,000 to less than $100,000 (5)
- More than $100,000 (6)
- I would rather not say (7)

Which one best describes your family structure:

- 2 parents or caregivers/nuclear family in one household (1)
- Separated parents with dual custody (2)
- Step-family (3)
- Single caregiver (4)
- Other family structure (5) (add a box so they can describe)

Maternal Education:

- Completed graduate degree (1)
- Bachelor degree (2)
- Post-secondary diploma or trades certificate (3)
- Some college but did not complete (4)
- High school diploma (5)
- GED (6)
- Did not complete high school (7)
- Unknown (8)
- Not applicable (9)

Paternal Education:

- Completed graduate degree (1)
- Bachelor degree (2)
- Post-secondary diploma or trades certificate (3)
- Some college but did not complete (4)
- High school diploma (5)
- GED (6)
- Did not complete high school (7)
- Unknown (8)
- Not applicable (9)

Primary Income earner’s job:

Primary income earner’s employment status:

- Full-time (1)
- Part-time (2)
- Unemployed (3)
- Pension (4)

End of Block: Demographic Information

Start of Block: Final Questions

What do you think are the best areas for resource allocation to better support children and families impacted by movement difficulties? (e.g., more resources for families, increased awareness and support by teachers)?

________________________________________________________________

________________________________________________________________

________________________________________________________________

________________________________________________________________

________________________________________________________________

What area of support is the highest priority for you at present?

________________________________________________________________

________________________________________________________________

________________________________________________________________

________________________________________________________________

________________________________________________________________

Are there any other comments or concerns you would like to raise?

________________________________________________________________

________________________________________________________________

________________________________________________________________

________________________________________________________________

________________________________________________________________

Do you wish to find out more information about the DCD Research Database? This is a database developed by Dr. Jill Zwicker providing contact information about future research projects. If yes, please provide your name and email address. Your survey responses to this questionnaire would remain confidential and de-identified, and your contact information would not be linked to the survey responses. Providing your name and email would only be used to send **information** about the DCD Research Database. A separate consent process to register with the database would be required, if you provide us with your contact details.

Name: ___________________________

Email Address: ________________________

Thank you for taking the time to complete this survey

End of Block: Final Question
